# Characterization and diagnostic marker for *TTG1* regulating tannin and anthocyanin biosynthesis in faba bean

**DOI:** 10.1038/s41598-019-52575-x

**Published:** 2019-11-07

**Authors:** Natalia Gutierrez, Ana M. Torres

**Affiliations:** Área de Genómica y Biotecnología, IFAPA-Centro Alameda del Obispo, Apdo 3092, E-14080 Córdoba, Spain

**Keywords:** Agricultural genetics, Gene expression profiling

## Abstract

Condensed tannins, found in coloured-flowering varieties of faba bean (*Vicia faba* L) are, after vicine and convicine, one of the major anti-nutritional factors for monogastric animals. The development of tannin-free cultivars is a key goal in breeding to broaden the use of this legume in the animal feed industry. Two recessive genes, *zt-1* and *zt-2*, control the zero-tannin content and promote white-flowered plants. Previous studies exploiting synteny with the model *Medicago truncatula* reported a mutation in *TTG1*, a gene encoding a WD40 transcription factor located in chromosome II, as the responsible for the *zt-1* phenotypes. Here a comprehensive analysis of *VfTTG1* (including phylogenetic relationships, gene structure and gene expression) has been conducted to confirm the identity of the gene and to reveal structural changes that may result in different functional alleles. The results confirmed the identity of the candidate and revealed the existence of two different alleles responsible for the phenotype: *ttg1-a*, probably due to a mutation in the promoter region, and *ttg1-b* caused by a deletion at the 5′end of *VfTTG1*. Based on the sequencing results, an allele-specific diagnostic marker was designed that differentiate zt-1 from wild and zt-2 genotypes and facilitates its deployment in faba bean breeding programs.

## Introduction

Faba bean (*Vicia faba* L.) is a high nutritional legume crop, traditionally used for animal feeding and human consumption. With seeds rich in protein (from 247 to 372 g/kg DM) and energy (15.6 MJ/kg DM), their ability to fix free nitrogen and to grow in different climatic zones, faba bean today is a prominent food grain legume in the world^1^. Despite its agronomic, nutritional and potential health benefits, the crop is still underutilized in South and South-East Asian countries^2^ and not fully exploited in higher-economy countries, where meat is the major source of protein in the diet. Nevertheless, the potential of faba bean as a protein-rich fodder crop is constrained by a number of factors such as the tannins, which are primarily located in the seed coat^3,4^.

Condensed tannins and anthocyanins are major flavonoid end-products of a well conserved family of aromatic molecules that fulfil a large number of biological functions in plant development and defence. For instance, anthocyanin pigments are visual signals to pollinators and contribute to flower, fruit and seed colours. Likewise, flavonoids play multiple roles in plant protection against fungal pathogens, insect pests and larger herbivores^5–10^. Both compounds are produced by related branches of the flavonoid pathway and utilize the same metabolic intermediates.

Despite these protective effects, tannins have a negative impact on nutritional value and digestibility of monogastric animal, being responsible for a decrease in feed intake, growth rate, feed efficiency, net metabolizable energy, and protein digestibility^3,4,11^. Consequently, the development of tannin-free cultivars is a key breeding objective to enhance the nutritional quality and broaden the use of this legume crop in livestock feeds industry.

In faba bean the zero tannin phenotype is under monogenic control of two complementary and recessive genes, *zt-1* and *zt-2*^3^. Both mutations interrupt the anthocyanin biosynthetic pathway at different steps, resulting in zero tannin and white flowered phenotypes^4,12^. For this reason, the allogamy present in the species is a major challenge to produce tannin free varieties. Crosses of white flowered plants with wild types or between lines carrying different zero tannin genes during multiplication can occur, resulting in coloured F_1_ plants with tannins that contaminate the seed lots. Thus, it is important to maintain careful isolation among phenotypes and to characterize the genes present in each of the genebank tannin-free accessions to assist breeders in the selection of appropriate genitors^13^.

Transcription factors (TFs) are proteins that bind to the DNA of promoter or enhancer regions in target genes to modulate the rate of gene transcription. Studies of plant mutants have revealed that informative phenotypes are often caused by mutations in genes for TFs that act as key regulators of various plant functions^14^. The anthocyanin biosynthetic pathway has been well characterized in many plant species. The process is transcriptionally regulated by the MYB-bHLH-WD (MBW) complex^15–17^ mainly controlled by TT2 (Transparent Testa 2), a member of the MYB family of TFs; TT8 (Transparent Testa 8), a basic helix–loop–helix (bHLH) TF; and TTG1 (Transparent Testa Glabra 1), a WD40 protein which together form the ternary MBW complex. The WD40 domain, generally composed of seven repeats or multiples of seven^18^, is one of the most abundant and interacting domains in eukaryotic genomes. Anthocyanin transcriptional regulators differ between monocot and dicot species. In monocots such as the maize, genes are activated by the MBW complex while in dicots, as faba bean, early biosynthesis genes are activated by independent *R2R3*-MYB TFs, while genes encoding functions in the anthocyanin pathway are activated by the MBW in the late steps^19^. In flowers of pea and lentils the absence of pigmentation is the result of a mutation in the *bHLH* regulatory gene leading to a mis-spliced mRNA causing a premature stop codon^20,21^. By contrast, in *Arabidopsis thaliana*^22^ and *Medicago truncatula*^23^, *TTG1* is an essential regulator of late structural genes in flavonoid biosynthesis.

Combining genetic mapping and the known synteny with the model *M*. *truncatula*, a similar result has been reported in faba bean, where an ortholog of the *Medicago* WD40 transcription factor *TTG1*, located in chromosome II, was identified as the gene responsible for the *zt-1* phenotypes^24,25^. A deletion in the recessive allele sequence plausibly explained the *zt-1* unpigmented phenotype^25^. However, complementary analyses are required to confirm the identity of the candidate gene and to clarify if other possible structural changes in this gene result in distinct non-functional alleles.

In this study a comprehensive gene analysis including phylogenetic relationships, gene structure and gene expression was carried out, to characterize all the *TTG1s* in faba bean. Our analyses have been focussed on the *TTG1* sequences to determine the evolutionary relationships between the *TTG1* gene of faba bean (*VfTTG1*) and those in other species. In addition, the expression profile in immature and young faba bean petals was examined to reveal a key role of *VfTTG1* in the regulation of the flavonoid biosynthesis. Finally, to help breeders in the selection of appropriate genitors, our aim was to develop a diagnostic marker efficient in the selection of white flowered cultivars carrying the *zt-1* gene, free of tannins. Diagnostic markers, also known as perfect markers, are directly linked with the allele of the locus of interest and recombination between gene and marker is absent. This avoids false selection or loss of information in marker-assisted breeding, thereby allowing a more efficient and accurate screening for allelic diversity in large germplasm collections^26–28^.

In summary, the objectives of this study were: (i) to provide a detailed molecular characterization of the allele(s) at the *zt-1* locus, (ii) to confirm *Transparent Testa Glabra* (*TTG1*) as the gene responsible for a loss of pigmentation in flowers and absence of tannins in faba bean seeds and (iii) to develop an allele-specific diagnostic marker useful for faba bean breeding programs.

## Results

### Sequence analysis of *VfTTG1* gene

**Figure 1:**
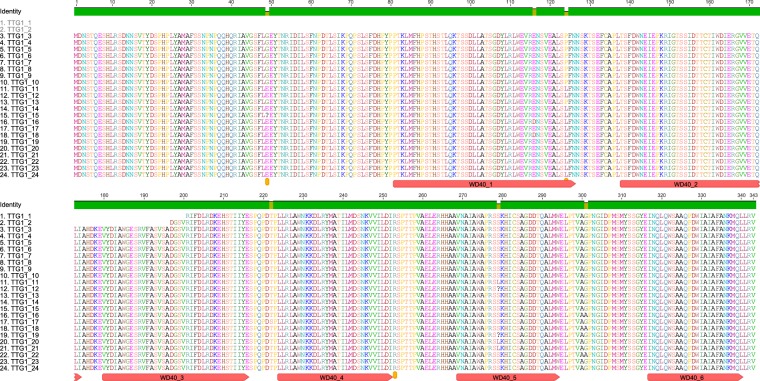
Alignment of the VfTTG1 amino acid sequences from the 24 faba bean lines used in this study differing for tannin content. The position of the six conserved domains, as well as of the three SNPs found in the transcribed sequence are indicated. I dentity corresponds to the faba bean lines listed in Table 2.

**Figure 2:**
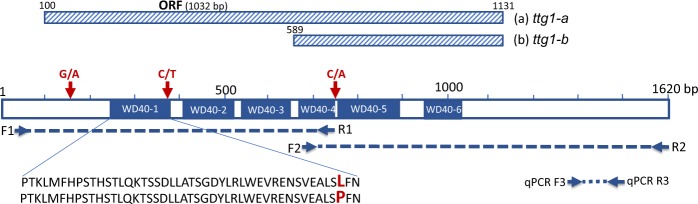
Genomic organization of the *VfTTG1* gene. Solid boxes represent the six conserved WD40 domains and the striped boxes a and b the two open reading frames (ORF) amplified in this work, giving rise to two distinct alleles (*ttg1-a* and *ttg1-b*). The position of the primer pairs used in the study is indicated by arrows and the corresponding PCR products in dashed lines. The three SNPs find between the wild and the zt1 types, are indicated with an inverted arrow. The SNP present in the conserved domain WD40-1, results in the substitution of a highly conserved leucine residue with a proline.

Primers pairs derived from the faba bean contig2007 (Table 1), were used to amplify and sequence the *M*. *truncatula TTG1* ortholog in faba bean. The full length *VfTTG1* gene, detected in most of the 24 lines assayed, was of 1605 bp (Supplementary Fig. S1) and the open reading frame (ORF) of 1032 bp, encoding a predicted protein of 343 amino acids. Lines ALBUS and EB0T0V contained a shorter ORF of 143 and 147 amino acids, respectively (Fig. 1) due to a large deletion in the 5′ region of the gene. Unexpectedly, the *VfTTG1* nucleotide sequence in zt1 (line 3) and one of the zt2 genotypes (line 13) were identical in contrast to the other zt2 and wild accessions (Table 2). Sequence alignment between the different lines revealed three single nucleotide polymorphisms (SNPs) (Fig. 1). The first SNP (G/A, being G present in the zt1 lines), located 244 bp downstream of the nucleotide sequence, generates an amino acid change from Glycine in lines *zt1* to Glutamic acid in the rest of lines. The second substitution (C/T), at position 469 bp, results in a change from Proline in lines zt1 to Leucine in the rest of lines. This mutation is located within the highly conserved domain WD40-1. The third SNP (C/A), at position 855 bp, has no effect on the amino acid sequence. In contrast to the rest of lines, and due to the deletion in the 5′end of the gene, amplification of *VfTTG1* in EB0T0V and ALBUS was only possible with the second primer pair, Vf_TTG1-F2/R2 (Table 1; Figs 1, 2 and S1).

**Table 1:** Primer sequences used for the amplification of *VfTTG1* and reference genes *CYP2* and *ELF1A*, for the normalization of the RNA expression. Position in the genome and product amplicon sizes are indicated.

Primer pair	5′-3′ Nucleotide sequence	Position in faba bean genomic DNA	Lenght of PCR product
Vf_TTG1-F1/R1	F1: TATGAATTCATTTTTAGTTCCCACCTAAC	10 to 29	754 pb
	R1: GTATCCGGTTGAGGACTCTCATAGATA	737 to 763	
Vf_TTG1-F2/R2	F2: GATGGGTCTGTTAGGATCTTTGATTT	687 to 712	934 pb
	R2: ATACAACTAACTTCATTGACTAAATATCTAC	1590 to 1620	
Vf_TTG1-F3/R3 (qPCR)	F3: TGCAATGGCTTCATATGTAAGAGTG	1282 to 1306	107 pb
	R3: CAACAAGCAAATGCAACATCAATTTC	1363 to 1388	
CYP2-F/R	F: TGCCGATGTCACTCCCAGAA	Reference genes for qPCR (Gutierrez *et al*. 2011)	
	R: CAGCGAACTTGGAACCGTAGA		
ELF1A-F/R	F: GTGAAGCCCGGTATGCTTGT		
	R: CTTGAGATCCTTGACTGCAACATT

**Table 2:** List of the faba beans lines tested in this study.

ID_seq	Faba bean lines	Tannin content^*^
1	ALBUS^a^	zt1
2	EB0T0V^d^	zt1
3	zt1 PARENTAL LINE^b^	zt1
4	GLORIA 5^c^	zt1
5	POLLEN 2074^d^	zt1
6	BLANDINE 2073^d^	zt1
7	OPTICA 1482^d^	zt1
8	FABIOLA OT 2318^d^	zt1
9	19 TB OT 2316^d^	zt1
10	GLORIA 2308^d^	zt1
11	DISCO 2390^d^	zt2
12	F2 (WXD)^d^	zt2
13	zt2 PARENTAL LINE^b^	zt2
14	WIZZARD^d^	T
15	VF6 PARENTAL LINE^b^	T
16	DIVA 2366^d^	T
17	FABIOLA T 2319^d^	T
18	19 TB T 2317^d^	T
19	LADY 2401^d^	T
20	MELODIE 2393^d^	T
21	DIVINE 2391^d^	T
22	G 58 MAINTENEUR 2302^d^	T
23	AD 23 MAINTENEUR 2300^d^	T
24	MAYA^d^	T

^*^T: genotype with tannins. zt1: genotype with lack of tannins carrying the *zt-1* gene. zt2: genotype with lack of tannin carrying the *zt-2* gene.

^a^Lines provided by Dr. O’Sullivan (U. Reading).

^b^Lines provided by IFAPA.

^c^Lines provide by Dr. Link (U. Gottingen).

^d^EUCLEG project lines provided by Dr. Duc (INRA).

### Bioinformatics analysis of *VfTTG1* gene

The 1032-bp ORF of *VfTTG1* encodes a predicted protein of 343 amino acid residues with a molecular weight of 36.76 kDa. The protein’s formula is C_1710_H_2634_N_464_O_530_S_12_. and the instability index (50.22), classifies the protein as unstable. The hydrophilic amino acid distribution of *VfTTG1* is relatively uniform and the number (54%) is higher than that of hydrophobic amino acids. The secondary structure of VfTTG1 reveals a 45 α-helix (13.12%), 166 random coil (48.40%), 115 extended peptide chain (33.53%), and 17 β-turn (4.96%). The phosphorylation site prediction results revealed 29 Ser, 12 Thr and 5 Tyr phosphorylation sites that may participate in phosphorylation control regulating and modulating protein-protein binding.

VfTTG1 showed homology to the WD40 superfamily including six WD40 repeats units (Figs 1 and 2). Sequence alignment with WD40 proteins from other legume species revealed a high conservation among the repeated sequences. Thus, *Pisum sativum*, *Cicer arietinum*, *M*. *truncatula*, *Trifolium pratense*, shared 97.38%, 95.34%, 92.71% and 92.13% amino acid identity, respectively, with *VfTTG1*. Sequence identity was slightly lower in the case of *Lotus corniculatus* (88.86%), *Phaseolus vulgaris* and *Vigna unguiculata* (82.80%) and *Glycine max* (81.34%) reaching 79.13% when compared with the non-legume species *Arabidopsis thaliana*.

**Figure 3:**
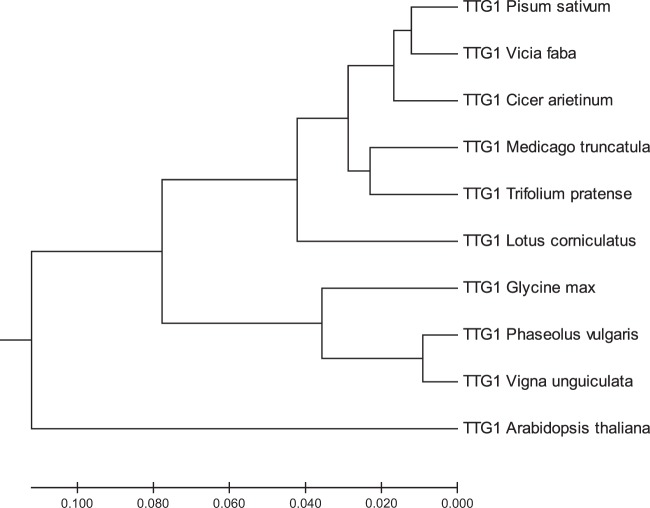
Phylogenetic tree analysis of TTG1 proteins from faba bean (*Vicia faba* L.) and other plant species. The analysis was based on amino acid homology. The ten accessions sequences used were: *P*. *sativum* (ADQ27310), *C*. *arietinum* (XP_004502764), *M*. *truncatula* (XP_003602392), *T*. *pratense* (PNX92996), *L*. *corniculatus* (ARK19314), *G*. *max* (XP_003523314), *P*. *vulgaris* (XP_007136431), *V*. *unguiculata* (XP_027942759) and *A*. *thaliana* (NP_197840). The optimal tree with the sum of branch length = 0.46937337 is shown. The tree is drawn to scale, with branch lengths in the same units as those of the evolutionary distances used to infer the phylogenetic tree. Evolutionary analyses were conducted in MEGA7.

The phylogenetic tree based on amino acid sequences of *VfTTG1* and *TTG1s* from other species produced two well defined clusters of *TTG1s*, distinguishing legume species and Arabidopsis and supporting the close phylogenetic relationships among the Fabaceae sequences as compared with the Brassicaceae. According with the percent identity observed in the *VfTTG1* sequences, the legumes were divided in two major groups corresponding to the Galegoid (*V*. *faba*, *P*. *sativum*, *C*. *arietinum*, *M*. *truncatula*, *T*. *pratense* and *L*. *corniculatus*) and Phaseoloid (*G*. *max*, *V*. *unguiculata* and *P*. *vulgaris*) species. *VfTTG1* showed the closest relationship with the *P*. *sativum* sequence. Both species belong to the tribe Viceae and grouped into a cluster (close to *C*. *arietinum*), while TTG1s from *Medicago* and *Trifolium* belonging to the Trifoliae grouped into a different cluster. Members from these two tribes, together with the genus *Lotus*, fall together into the Galeloid clade, often referred to as cool season legumes. Finally, *TTG1s* from the Phaseoloid species group together in a separate clade corresponding to the tropical season legumes respectively (Fig. 3).

### Expression analysis of *VfTTG1* gene

The dissociation curve analysis confirmed that the primer pair Vf_TTG1-F3/R3, produced a single and specific PCR product. The mean PCR efficiency for *VfTTG1* was 94.8%, while for *ELF1A* and *CYP2* were 93.3% and 92.5%, respectively.

Supplementary Fig. S2 provides an overview of the transcript levels of genes *VfTTG1*, *CYP2* and *ELF1A*. The expression levels of these genes in Vf6 (line 15), zt1 (line3) and zt2 (line 13), in two stages (S1 and S2), were determined as quantification cycle (Cq) values. Transcripts abundance varied widely thus, the reference genes showed the lowest Cq values in all samples and stages (S1 and S2) stages while the average Cq values for *VfTTG1* ranged from 21.29 in Vf6 line to 34.58 in zt1 line (Supplementary Fig. S2). Zt1 showed the highest Cq values in all stages suggesting a clear down regulation of the corresponding transcript.

**Figure 4:**
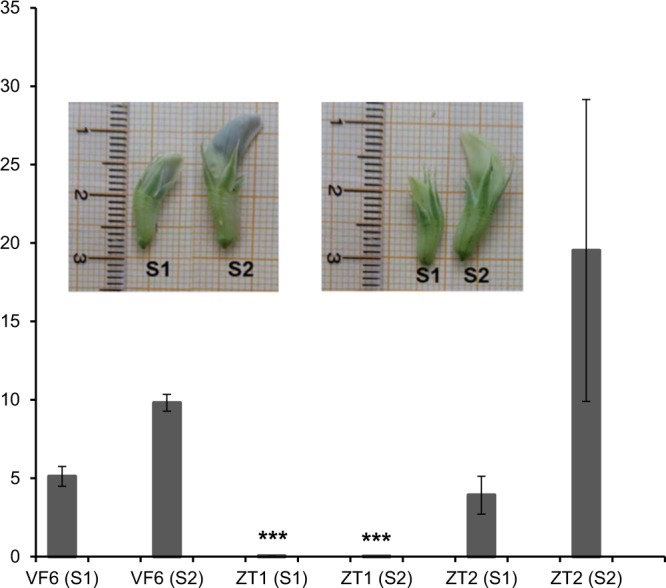
Bar graph representing the expression profiles of *VfTTG1* in the wild flowered line Vf6 and in the white flowered zt1 and zt2 lines, at different developmental stages (S1 and S2). Relative mRNA quantification was performed using *CYP2* and *ELF1A*, as reference genes for normalization. Transcripts levels were analysed in two biological replicates. ***Denotes P < 0.001 statistically highly significant vs the other samples (ANOVA test). The error bars indicate the SEM (standard error of the mean).

The *VfTTG1* expression profiles for each parental line (Vf6, zt1 and zt2) and flower developmental stage are shown in Fig. 4. Similar expression patterns were observed in Vf6 and zt2 as compared with zt1. Using the ANOVA test, we find a highly significant expression level difference (p < 0.001) in the zt1 expression with respect to the rest of genotypes. These results further demonstrate that *Transparent Testa Glabra 1* (*TTG1*) is the gene responsible for the loss of flower pigmentation and the absence of tannins in zt1 genotypes.

### Allele-specific diagnostic marker

**Figure 5:**
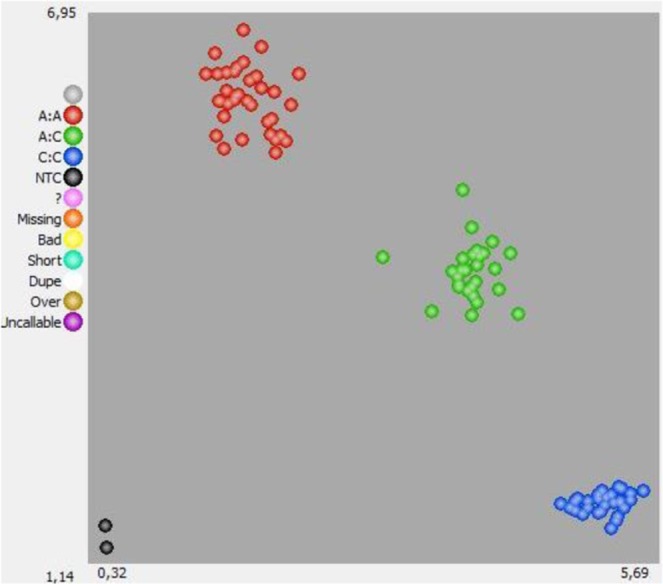
KASP genotyping results of marker KASP_TTG1 in the two faba bean sets (**A**,**B)**. The scatter plot with axes x and y shows the allelic discrimination of the accessions that clustered into three different groups: FAM homozygotes (blue), HEX homozygotes (red), and FAM/HEX heterozygotes (green). No-template controls (NTC). Allele “A”, in red, co-segregates with wild and zt2 genotypes while allele “C”, in blue, corresponds to the zt1 haplotype and line 13.

Allele C corresponded to the *zt-1* haplotype which co-segregated with all the zt1 genotypes while the allele A co-segregated with wild and zt2 genotypes. Only line 13, one of the zt2 genotypes showing identical *VfTTG1* sequence to zt1, deviated from this pattern. The detailed genotypic and phenotypic data for both sets of lines (A and B) are presented in Supplementary Table S1 and S2, respectively. The phenotypic data linked to each of the possible alleles (C vs. A) showed a clear distinction between individuals for TC (Fig. 5). Thus, KASP_TTG1 confirmed the known phenotypes in all the set A lines assayed and provided a reliable tool for calling heterozygous genotypes. In set B, the segregation of the KASP_TTG1 marker also matched with the expected phenotypes for flower colour and TC. Moreover both, homozygous and heterozygous genotypes were correctly predicted (Supplementary Tables S1 and S2). These results demonstrate that the KASP_TTG1 marker identifies unequivocally unpigmented faba bean genotypes carrying the *zt-1* gene.

## Discussion

Unlike for ruminants, tannins have traditionally been considered as antinutritional factors in monogastric nutrition with negative effects on feed intake, nutrient digestibility and production performance^29^. For these reasons, the development of faba bean tannin-free cultivars is a key breeding objective for the livestock feed industry. Tannins share some precursors in their biosynthetic pathway with anthocyanin pigments, which explains the well-known relationship between white flowered plants and absence of tannins in their seeds^30^. Two complementary recessive genes, *zt-1* and *zt-2*, disrupt the synthesis of anthocyanins at different steps, resulting in white flowered plants lacking tannins.

In two previous studies^24,25^, a combination of genetic linkage, association studies and functional and comparative genomics was used to determine which putative enzymes or transcriptional regulators of the flavonoid pathway could putatively be encoded by the *zt-1* gene. After identifying the syntenic regions between faba bean and *Medicago* the search of regulatory genes pointed towards the transcription factor *TTG1* (*Transparent Testa Glabra 1*) as responsible for the loss of floral pigmentation in the two white flowered lines analysed, zt1 and ALBUS. Moreover, the results reported by^25^, indicated that white pigmentation in line ALBUS is due a deletion in the *VfTTG1* coding region. *TTG1* is a WD40 repeat protein broadly involved in plant regulation of seed coat development and pigmentation, trichome formation in leaves and of flavonoid and anthocyanin biosynthesis pathway. Mutations in *TTG1* affect anthocyanin production in both seeds and vegetative parts^31^.

In this study a comprehensive analysis of *VfTTG1*, including phylogenetic relationships, gene structure and gene expression, was conducted to confirm its identity and to clarify if other structural changes in the gene result in different non-functional alleles. First, the *M*. *truncatula* WD40 amino acid and protein sequences were used to identify the corresponding ortholog in the faba bean transcriptome^32^. Transcriptome mining identified contig2007, whose sequence was used to design two primer pairs (Table 1; Fig. 2). These primers were used to assay 24 faba bean lines (Table 2), and the PCR products were sequenced.

All the tested faba bean lines, except EB0T0V and ALBUS, produced two overlapping DNA fragments that together corresponded to the full length transcript of 1605 bp and to the ORF of 1032 bp. EB0T0V and ALBUS carry a deletion encompassing the 5′ end of *VfTTG1* which was previously shown to be responsible for the loss of floral pigmentation^25^. Our results reveal, for the first time, the presence of two non-functional allelic forms of the *VfTTG1* gene. The predominant allele (*ttg1-a*), corresponds to the longer ORF (1032 bp) and is present in the white flowered lines 3 to 10 while the other allele (*ttg1-b*) is only found in lines 1 and 2 (Table 2; Fig. 2).We thus investigated the possible reasons for the loss of flower colour in lines 3 to 10. Although sequence alignment revealed the presence of three SNPs in the transcribed sequences, two missense and one silent mutation (Figs 1 and 2), none of these appears to be causative of the TTG1 loss of function.

Measurement of *VfTTG1* expression in flowers from the parental lines Vf6, zt1 and zt2, at two development stages, revealed similar expression levels in the wild parental Vf6 and zt2 lines and lack of expression in the zt1 genotype (Fig. 4). This result suggests that the causative mutation in zt1 must lie in the promoter or 5′-UTR region affecting either gene expression or the transcript stability. Nevertheless, further analysis should be performed to confirm this hypothesis. Despite the contrasting expression patterns revealed by the white genotypes zt1 and zt2, both lines showed identical *VfTTG1* nucleotide sequence. To discard possible errors in sequencing or a DNA contamination the sequence analysis was repeated in 15 different zt2 plants (data not shown) but the result was confirmed. These facts corroborate the already known control of the trait by two independent and complementary recessive genes *zt-1* and *zt-2*, which are located in different chromosomes^3,33^. We are currently combining genetic linkage^34^ and synteny-based candidate gene discovery to saturate the genomic region carrying *zt-2* and to identify candidates associated with this trait. The *zt-2* syntenic region is in the distal part of chromosome III, tightly linked to GLIP245SNP (MTR_1g071430)^35^. After this initial linkage finding, fine mapping is being carried out to narrow down the putative *zt-2* locus.

The *VfTTG1* amino acid sequence was used to identify conserved domains, analyse the physical and chemical parameters and predict putative phosphorylation sites. WD40 proteins are characterized by 4–16 tandem repeats of a conserved peptide motif of 40–60 amino acids^36–39^. A typical WD40 domain contains seven or multiples of seven repeats forming a highly stable β-propeller structure^38^. The faba protein coding sequence included six WD40 repeating units of a motif of about 40 amino acids (Figs 1 and 2). In Arabidopsis, the WD40 repeat protein *TTG1* is vital for different aspects such as epidermal cell fate and positively regulates trichome formation, anthocyanin production, seed-coat pigmentation and seed-coat mucilage production while negatively regulating hypocotyl stomatal-cell identity and root-hair formation^23,38^. In faba bean as well as in *Medicago*, the WD40 repeat protein is necessary for tissue-specific anthocyanin and proanthocyanidin biosynthesis^23^.

The phylogenetic tree based on the *TTG1* amino acid sequences revealed two defined clusters, Fabaceae and Brassicaceae. While *VfTTG1* showed the closest relationship with *P*. *sativum*, all legume species grouped together in two main clades, Galegoid and Phaseoloid, reflecting the common evolutionary origin of these taxa.

In this study we have provided further evidence suggesting that *VfTTG1* encoding a WD40 transcription factor is located at the *zt-1* locus in faba bean and that this WD40 gene has at least two distinct alleles, *ttg1-a* and *ttg1-b*. The *ttg1-a* allele in line zt1 carries a mutation in the 5′ region, affecting the gene expression or the transcripts stability, while the *ttg1-b* allele, has a deletion encompassing the 5′ *VfTTG1* end. These findings were here supported by qPCR analyses, showing that *VfTTG1* was not expressed in zt1, in contrast to the high expression in floral buds of wild and zt2 genotypes.

A diagnostic molecular marker (KASP_TTG1) using the competitive allele specific PCR (KASPar) assay has been developed to differentiate *zt-1* from wild and *zt-2* alleles. Except for line 13, KASP_TTG1 was validated in all the wild and zt2 lines tested, including heterozygous genotypes, thus providing a reliable and accurate tool for marker-assisted selection of TC in faba bean breeding programmes (Fig. 5). Additional characterization of the *VfTTG1* promoter region is required to provide a perfect diagnostic marker for *zt1*. Further research on genes involved in faba bean flower and seed pigmentation will increase our understanding of the regulation of this biosynthetic pathway and likely to favour the development of new tools for the generation of value-added faba bean cultivars with optimized flavonoid content.

## Material and Methods

### Plant material and sample collection

Eleven lines of faba bean with wild-type flowers and high tannin content (TC), ten *zt*-1 and three *zt*-2 mutant genotypes with white flowers and zero TC (Table 2), were grown under insect-proof field cages to avoid cross-pollination. Young leaves were collected from all samples and stored at −80 °C. DNA was isolated according to^40^.

### Primer design, PCR optimization and sequencing

The *M*. *truncatula TTG1* (MTR_3g092840, AES72643) gene sequence was used as a query against the protein databases of the National Center for Biotechnology Information (NCBI) to retrieve the orthologous sequences in related species. We also searched the corresponding faba bean sequence among the in-house faba bean transcriptome data^32^. Contig2007 sharing more than 86% identity with the query sequence, was used to design a set of primer pairs in conserved regions (Table 1, Fig. 2). Sequence alignment and primer design were performed using Geneious v.7.1.9. (https://www.geneious.com). PCR reactions were performed in a total volume of 25 µl, using 5 µl DNA of each sample, 200 nM of each primer, 2 mM MgCl_2_, 200 µM dNTP and 0,6 U Taq polymerase Biotools (B&M Labs, S.A., Madrid, Spain). The annealing temperature used for amplification of primer pairs was 60 °C.

The PCR amplification products from 24 faba bean lines were purified using a standard protocol (https://www.thermofisher.com) for DNA precipitation with sodium acetate and ethanol (1/10 3 M sodium acetate, 2 v/v ethanol). PCR products were sequenced by Sanger at STABVIDA (Caparica, Portugal). For each sample, four PCRs were done, and the repeated products were then mixed for sense and antisense strand sequencing.

### Bioinformatic analysis

Based on the *M*. *truncatula* sequence, the presence of putative open reading frames (ORF) in the new sequences was predicted. Conserved domains were identified by comparison against the NCBI Conserved Domain Database Search against database CDD v3.17 (https://www.ncbi.nlm.nih.gov/Structure/cdd/wrpsb.cgi). The ExPASy-ProtParam tool was used to compute the physical and chemical parameters of the VfTTG1 protein (https://web.expasy.org/cgi-bin/protparam/protparam). The phosphorylation sites were predicted using ExPASy-NetPhos (http://www.cbs.dtu.dk/services/NetPhos/). BLASTX (NCBI) was applied to study the homology among the nucleotide sequences and to conduct multiple sequence alignment using Geneious.

A phylogenetic tree was constructed with 10 amino acid sequences from eight legume species related to *V*. *fab*a together with *Arabidopsis thaliana*. Sequences were searched by BLASTP (NCBI) using the VfTTG1 protein as a query and aligned by ClustalX. A phylogenetic tree was constructed by the UPGMA method^41^ using the MEGA7 software^42^. The evolutionary distances were computed using the Poisson correction method^43^ and displayed in the units of the number of amino acid substitutions per site. Positions containing gaps and missing data were eliminated and the final data set included 334 positions.

The GenBank accession numbers for the 24 nucleotide sequences analysed in this study were: TTG1_zt2 (MN119527), TTG1_zt1 (MN119528), TTG1_WIZZARD (MN119529), TTG1_Vf6 (MN119530), TTG1_MAYA (MN119531), TTG1_GLORIA (MN119532), TTG1_POLLEN (MN119533), TTG1_BLANDINE (MN119534), TTG1_OPTICA (MN119535), TTG1_DISCO (MN119536), TTG1_DIVA (MN119537), TTG1_FABIOLA_T (MN119538), TTG1_FABIOLA_OT (MN119539), TTG1_19_TB_T (MN119540), TTG1_LADY (MN119541), TTG1_MELODIE (MN119542), TTG1_DIVINE (MN119543), TTG1_19_TB_OT (MN119544), TTG1_GLORIA_2308 (MN119545), TTG1_G_58_MAINTENEUR (MN119546), TTG1_AD_23_MAINTENEUR (MN119547), TTG1_E0T0V (MN119548) and TTG1_ALBUS (MN119549).

### Quantitative real-time PCR samples and conditions

The most important guidelines of the MIQE checklist^44^ were considered according to the practical approach for quantitative real-time PCR (qPCR) experiments proposed by^45^. The expression profile of *VfTTG1* in pigmented flowers (line 15, Vf6) and white flowers (line 3, zt1 and line 13, zt2; Table 2) at two different developmental stages was analysed. Stage 1 (S1) included immature flowers buds of approximately 1,5 cm length while the stage 2 (S2) included young flowers of 2 cm length and black colour apparent at the top of the petal in the wild types (Fig. 4). *CYP2* and *ELF1A*, previously reported as the most stable genes for normalization of the gene expression in the TC experiment were used as reference^46^.

Only petal tissue was used for total RNA extraction using RNeasy mini kit (Quiagen, Valencia, CA, USA), according to the manufacter’s instructions. In order to minimise variation in gene expression among individual plants and stages, petal tissue from three individuals were pooled for RNA isolation. Finally, two pools of biological replicates were used per individual genotypes. Each sample was frozen in liquid nitrogen and stored at −80 °C until RNA extraction. In short, the experimental design consisted in a total of 24 samples (3 parental lines × 2 stages of development × 2 technical replicates × 2 biological replicates). The resulting RNA was treated with DNase I, RNase-free (Fermentas) according to the manufacturer’s instructions.

cDNA of each sample was obtained by qScript^TM^ cDNA SuperMix (Quanta Biosciences) and diluted to a concentration of 20 ng/µL. A pooled sample comprising all samples considered in the experiment was included for each gene as inter-run calibrator to detect and correct inter-run variation. No-template controls were also included.

The qPCR was carried out using the iTaq^TM^ Universal SYBR^®^ Green Supermix on an ABI PRISM 7500 Real Time PCR System (Applied Biosystems, Foster City, CA, USA). Experiments were performed in 96-well optical reaction plates (P/N 4306737) with MicroAmp optical adhesive film (P/N 4311971) from Applied Biosystems. A master mix with total volume of 11 µL for each PCR run was prepared, containing 5 µL of diluted cDNA, 5 µL of iTaq^TM^ Universal SYBR^®^ Green Supermix (Bio-Rad, Hercules, California) and a primer pair with a concentration of 0.22 µM each one. The PCR conditions were 95 °C for 10 min followed by 40 cycles at 95 °C for 15 s and 60 °C for 1 min. The specificity of the amplifications was confirmed by unique and sharp peak melting curves of the PCR products.

### qPCR data analysis

PCR efficiency of each primer pair was determined for all samples by the LinRegPCR quantitative PCR data analysis program v.11.0^47^ using raw normalized fluorescence as input data. The relative gene expression (RGE) was calculated using the Eq. (1) ^48,49^, where RQ = E^∆Ct^, being E = PCR efficiency for each primer and Ct = the number of cycles needed to reach 0.2 arbitrary units of fluorescence. The two reference genes (RG) used for data normalization were *CYP2* and *ELF1A*^46^.

1 $$RGE=\frac{RQ{\rm{VfTTG1}}}{Geomean[RQRG]}$$

Significance values to determine differences of the expression of genes were obtained by the ANOVA test using the R programming language.

### Allele-specific diagnostic marker

A KASP on demand (KOD) assay (LGC Genomics, Hoddesdon, UK) has been designed to identify unpigmented genotypes carrying the *zt-1* gene (KASP_TTG1 marker). Nucleotide sequences of the *VfTTG1* gene from the 24 faba bean lines were aligned using Geneious and analysed for SNPs. Three SNPs 244, 469 and 855 nucleotides upstream from the *VfTTG1* STOP codon were detected. The SNP in the last position (855 bp), distinguishing zt1 from wild type was the one chosen to design the KASP assay (Supplementary Fig. S1). The allele specific primers were A1 = GGATAGTAATAAAGTTGTGATTTTGGATATTA and A2 = GGATAGTAATAAAGTTGTGATTTTGGATATTC and the conserved primer was C1 = ACAGCAGCATGATGCCTCTCCAATT. To validate the KASP assay, we used two sets of faba bean lines. Set A (Supplementary Table S1) included 49 accessions with different genetic backgrounds carrying different alleles in the genes controlling flower color and TC. Set B (Supplementary Table S2) consists of 33 F_2_ individuals, derived from the cross between MAYA, a wild-type flowered line with high TC and EB0T0V, a white flowered line carrying the *zt-1* gene. DNA was extracted from F_2_ individuals and parental lines using the DNeasy Plant Mini Kit (Quiagen, Valencia, CA, USA). The assay reaction volume was 10 µL, being 5 µL of DNA (35 ng/µL), 5 µL of 2X KASP Master Mix and 0.14 µL KASP Assay Mix (allele-specific primer, A1 and A2, and common primer, C1). A. The PCR for KASP marker assay was set at 94 °C for 15 min, followed by a 36 cycle of 94 °C for 20 s and 57 °C for 60 s, then followed by 1 cycle of 30 °C for 60 s. Genotyping data were visualised using the SNPviewer PC tool.

## Supplementary information

Supplementary information

## Data Availability

The datasets generated and analyzed during the current study are available from the corresponding author on request.
