# Supplementary material for: Characterization and diagnostic marker for TTG1 regulating tannin and anthocyanin biosynthesis in faba bean
Source: Sci Rep. 2019 Nov 7;9:16174. doi: 10.1038/s41598-019-52575-x (PMC6838129; doi:10.1038/s41598-019-52575-x)
Supplement: Supplementary file 1 — Supplementary information [file 41598_2019_52575_MOESM1_ESM.pdf]

Characterization and diagnostic marker for *TTG1* regulating tannin and anthocyanin biosynthesis in faba bean

Natalia Gutierrez[1*], Ana M. Torres[1]
[1] Área de Genómica y Biotecnología, IFAPA-Centro Alameda del Obispo, Apdo 3092, E-14080 Córdoba, Spain.
*Corresponding author e-mail: natalia.gutierrez.leiva@juntadeandalucia.es

Supplementary Fig. S1 Multiple alignments of the VfTTG1 sequences from 24 faba bean lines Consensus identity corresponds to the faba bean lines listed in Table 2.

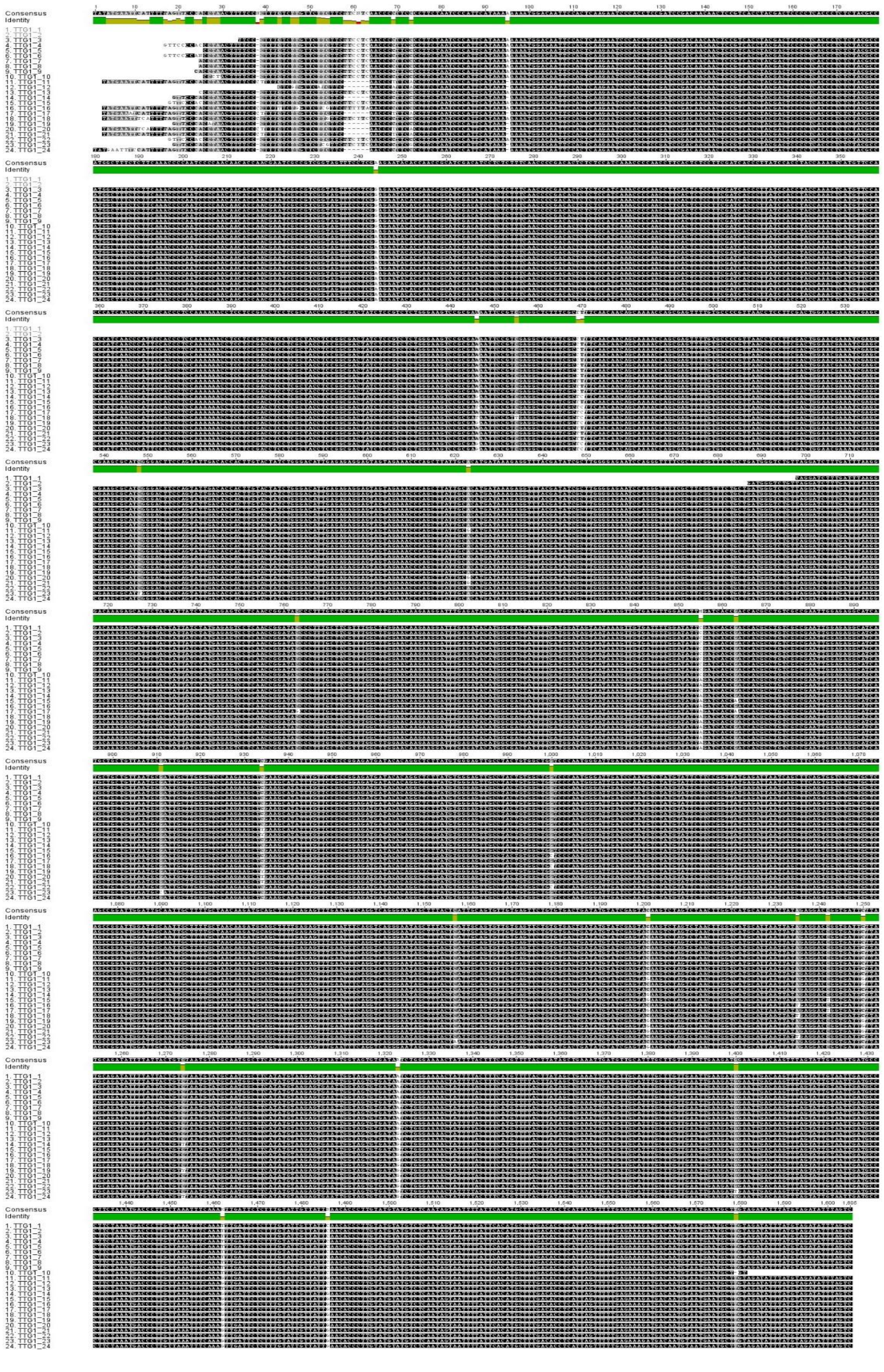

Characterization and diagnostic marker for *TTG1* regulating tannin and anthocyanin biosynthesis in faba bean

Natalia Gutierrez[1*], Ana M. Torres[1]
[1] Área de Genómica y Biotecnología, IFAPA-Centro Alameda del Obispo, Apdo 3092, E-14080 Córdoba, Spain.
*Corresponding author e-mail: natalia.gutierrez.leiva@juntadeandalucia.es

Supplementary Fig. S2 Quantification cycle (Cq) values of *VfTTG1*, *CYP2* and *ELF1A* in the faba bean samples VF6 (line 15), ZT1 (line3), ZT2 (line 13) at two different developmental stages (stage1: immature flowers and stage2: young flowers). The expression levels are shown as median (lines), 25 to 75 percentile (boxes) and range (whiskers).

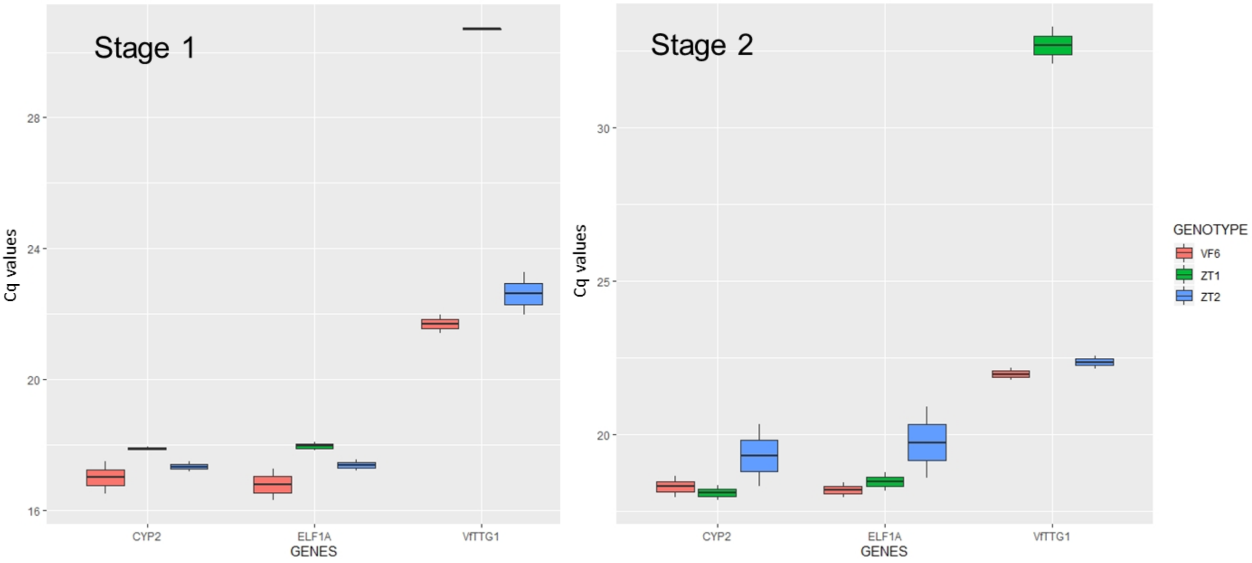

Characterization and diagnostic marker for *TTG1* regulating tannin and anthocyanin biosynthesis in faba bean

Natalia Gutierrez[1*], Ana M. Torres[1]

[1] Área de Genómica y Biotecnología, IFAPA-Centro Alameda del Obispo, Apdo 3092, E-14080 Córdoba, Spain.

*Corresponding author e-mail: natalia.gutierrez.leiva@juntadeandalucia.es

Supplementary Table S1. Phenotypes for tannin content/flower color and genotypes in the faba bean set A including 49 lines.

ID_lines	Faba bean lines	Phenotype[e]	Genotype[f]
1	ALBUS[a]	zt1	C:C
2	E0T0V[a]	zt1	C:C
3	AD 23 MAINTENEUR_2300[a]	T	A:A
4	G 58 MAINTENEUR_2302[a]	T	A:A
5	GLORIA_2308[a]	zt1	C:C
6	19 TB OT_2316[a]	zt1	C:C
7	DIVINE_2391[a]	T	A:A
8	MELODIE_2393[a]	T	A:A
9	LADY_2401[a]	T	A:A
10	19 TB T_2317[a]	T	A:A
11	FABIOLA OT_2318[a]	zt1	C:C
12	FABIOLA T_2319[a]	T	A:A
13	POUILLY_2327[a]	T	A:A
14	DIVA_2366[a]	T	A:A
15	DISCO_2390[a]	zt2	A:A
16	MAXIME_196[a]	T	A:A
17	AQUITAINE_267[a]	T	A:C
18	GERS_277[a]	T	A:A
19	LORRAINE_279[a]	T	A:A
20	PICARDIE_437[a]	T	A:A
21	DIANA_455[a]	T	A:A
22	MIKKO_1179[a]	T	A:C
23	TICOL_1191[a]	T	A:A
24	STRUBE_1216[a]	T	A:C
25	OPTICA_1482[a]	zt1	C:C
26	BOURDON_1505[a]	T	A:A
27	TROY_1579[a]	T	A:A
28	COTE D'OR_1626[a]	T	A:C
29	ASCOTT_1777[a]	T	A:A
30	SORAVI_2070[a]	T	A:C
31	BLANDINE_2073[a]	zt1	C:C
32	POLLEN_2074[a]	zt1	C:C
33	MAYA_2077[a]	T	A:A
34	PUNCH_2067[a]	T	A:C
35	WIZZARD[a]	T	A:A
36	GLORIA_5[a]	zt1	C:C
37	GL 101-04[b]	zt1	C:C
38	GL 102-04[b]	zt1	C:C
39	GL 103-04[b]	zt2	A:A

40	GL 105-04[b]	zt2	A:A
41	GL 106-04[b]	zt1	C:C
42	GL 107-04[b]	zt1	C:C
43	MXD_12[c]	zt2	A:A
44	MXD_13[c]	zt2	A:A
45	MXD_23[c]	zt2	A:A
46	WXD_51[c]	zt2	A:A
47	VF6[d]	T	A:A
48	ZT1[d]	zt1	C:C
49	ZT2[d]	zt2	C:C

[a] Origin : Line 1, provided by Dr. O´Sullivan (U. Reading); line 36, provided by Dr. Link (U. Gottingen); lines 2 to 35 derive from the EUFABA project and have been provided by Dr. Duc (INRA)

[b] Advances breeding lines provided by IFAPA

[c] White flowered F_2 individuals carrying the *zt-2* gene, derived from crosses between MAYA_2077 x DISCO_2390 (MxD) and WIZZARD x DISCO_2390 (WxD) and developed by IFAPA

[d] Parental lines provided by IFAPA

[e] T: genotype with tannin. zt1: genotype with zero tannin content carrying the *zt-1* gene. *zt2*: genotype with zero tannin content carrying the *zt-2* gene.

[f] Genotypic segregation of the KASP-TTG1 assay designed in this study

Characterization and diagnostic marker for *TTG1* regulating tannin and anthocyanin biosynthesis in faba bean

Natalia Gutierrez[1*], Ana M. Torres[1]
[1] Área de Genómica y Biotecnología, IFAPA-Centro Alameda del Obispo, Apdo 3092, E-14080 Córdoba, Spain.

*Corresponding author e-mail: natalia.gutierrez.leiva@juntadeandalucia.es

Supplementary Table S2. Phenotype for tannin content and flower color and genotype data in faba bean set B (33 F_2 individuals from cross MAYA_2077 x EB0T0V)

	ID_lines	Tannin[a]	Flower color[b]	Genotype[c]
1	MAYA_2077	T	A	A:A
2	EB0T0V	zt1	B	C:C
3	MxE063	T	H	A:C
4	MxE066	T	A	A:A
5	MxE067	zt1	B	C:C
6	MxE068	T	H	A:C
7	MxE069	T	H	A:C
8	MxE070	T	A	A:A
9	MxE071	zt1	B	C:C
10	MxE072	T	H	A:C
11	MxE073	T	H	A:C
12	MxE074	zt1	B	C:C
13	MxE075	zt1	B	C:C
14	MxE076	zt1	B	C:C
15	MxE077	T	H	A:C
16	MxE078	T	H	A:C
17	MxE080	T	H	A:C
18	MxE086	T	H	A:C
19	MxE088	T	H	A:C
20	MxE090	T	H	A:C
21	MxE091	T	H	A:C
22	MxE092	T	H	A:C
23	MxE093	T	H	A:C
24	MxE094	T	A	A:A
25	MxE096	T	H	A:C
26	MxE097	T	H	A:C
27	MxE098	T	A	A:A
28	MxE099	T	H	A:C
29	MxE100	zt1	B	C:C
30	MxE101	zt1	B	C:C
31	MxE103	T	H	A:C
32	MxE105	T	H	A:C
33	MxE106	T	A	A:A
34	MxE107	zt1	B	C:C
35	MxE108	T	A	A:A

[a] T: genotype with tannin. zt1: genotype with zero tannin content carrying the *zt-1* gene
[b] Phenotypic segregation of the flower color. (A): homozygous for normal flower (standard and keel petals white, wing petals white with a black spot); (B): homozygous for white flower; (H) heterozygous genotypes (spotted flower)
[c] Genotypic segregation of the KASP_TTG1 assay designed in this study
